# Model-Based Design of Growth-Attenuated Viruses

**DOI:** 10.1371/journal.pcbi.0020116

**Published:** 2006-09-01

**Authors:** Kwang-il Lim, Tobias Lang, Vy Lam, John Yin

**Affiliations:** Department of Chemical and Biological Engineering, University of Wisconsin–Madison, Madison, Wisconsin, United States of America; Max Planck Institute, Germany

## Abstract

Live-virus vaccines activate both humoral and cell-mediated immunity, require only a single boosting, and generally provide longer immune protection than killed or subunit vaccines. However, growth of live-virus vaccines must be attenuated to minimize their potential pathogenic effects, and mechanisms of attenuation by conventional serial-transfer viral adaptation are not well-understood. New methods of attenuation based on rational engineering of viral genomes may offer a potentially greater control if one can link defined genetic modifications to changes in virus growth. To begin to establish such links between genotype and growth phenotype, we developed a computer model for the intracellular growth of vesicular stomatitis virus (VSV), a well-studied, nonsegmented, negative-stranded RNA virus. Our model incorporated established regulatory mechanisms of VSV while integrating key wild-type infection steps: hijacking of host resources, transcription, translation, and replication, followed by assembly and release of progeny VSV particles. Generalization of the wild-type model to allow for genome rearrangements matched the experimentally observed attenuation ranking for recombinant VSV strains that altered the genome position of their nucleocapsid gene. Finally, our simulations captured previously reported experimental results showing how altering the positions of other VSV genes has the potential to attenuate the VSV growth while overexpressing the immunogenic VSV surface glycoprotein. Such models will facilitate the engineering of new live-virus vaccines by linking genomic manipulations to controlled changes in virus gene-expression and growth.

## Synopsis

The engineering of viral genomes provides ways not only to explore viral regulatory mechanisms at a genomic level, but also to produce recombinant viruses that may serve as vaccines, gene delivery vectors, and oncolytic (tumor-killing) agents. However, the complexity of interactions among viral and cellular components involved in the life cycle of a virus can make it challenging to anticipate how altering viral components will influence the overall behavior of the virus. Lim, Lang, Lam, and Yin have developed a computer model that begins to mechanistically account for key virus–cell interactions in its predictions of viral intracellular development. Lim et al.'s model was able to capture experimentally observed effects of gene rearrangements on the levels and timing of viral protein expression and virus progeny production, aspects that are important for the design of live-virus vaccines. Refinement and extension of their approach to current and other virus systems has the potential to advance the application of viruses as therapeutic agents.

## Introduction

Infections caused by viruses persistently threaten human health. For example, 40 million, 350 million, and 170 million people in the world are carrying human immunodeficiency virus type 1 (HIV-1), hepatitis B virus (HBV), and hepatitis C virus (HCV), respectively [1–3]. Annually 5% to 15% of the global population is infected with influenza, resulting in 250,000 to 500,000 deaths [4]. Protection against viral infections may be provided by inoculations with live-virus, killed-virus, or subunit vaccines. Live-virus vaccines offer advantages because they activate both humoral and cell-mediated immunity, require only a single boosting, and generally provide longer immune protection than other forms of vaccines. However, they must be adequately attenuated in their growth to minimize the possibility of vaccine-induced pathogenic effects while retaining their immunogenicity. Attenuation of live viruses has traditionally been achieved by serially passaging viruses in tissue or cell culture and adapting them to grow well on new cell types or at elevated or reduced temperatures [5], a process that tends to reduce their replicative ability and virulence in humans or animals [6]. Such attenuation has historically been a highly empirical process, where its mechanisms are often neither known nor elucidated.

During the last decade the emergence of reverse genetics techniques has created unprecedented opportunities to better control viral attenuation [7–9]. Reverse genetics enables the production of RNA viruses from cloned cDNA, so specific mutations can be relatively easily introduced into viruses. The challenge to engineering viruses for attenuation then shifts from creating the variants to predicting how specific genetic changes define or correlate with measurable effects on virus growth. Such a challenge can be addressed through the development of quantitative and mechanistic models that map genome-level changes to the dynamics of virus growth under different environmental conditions. Models of intracellular virus growth aim to predict how rapidly a virus-infected cell will produce virus progeny. Inputs to such models include rates of constituent processes such as entry of the virus into the cell, transcription of viral mRNAs, translation of viral proteins, replication of viral genomes, assembly of intermediates, and finally, production and release of viral progeny. Decades of detailed biochemical, biophysical, and genetic studies have, for diverse viruses, contributed toward a level of mechanistic understanding of viral functions and interactions. Various intracellular models of virus growth have been developed for phage Qβ [10], phage T7 [11,12], HIV-1 [13], and influenza A virus [14].

How can a detailed model for the intracellular growth of a virus be used to explore the behavior of mutant viruses that encode alternative designs? As a starting point, one can create alternative designs that reorder or rearrange the wild-type genes or regulatory elements. Such genomic changes can alter the timing and level of expression of different viral genes, and thereby impact growth because the production of viral progeny depends on the dynamic expression of viral genes. Preliminary models of such alternative genome designs can use the “language” of the wild-type virus. They retain the parameters that characterize wild-type molecular interactions, wild-type average rates of viral polymerase elongation, and wild-type composition of progeny viruses, but they apply them in a manner that reflects the reordering of wild-type components in the engineered genome. For example, the timing of expression for the genes of phage T7 during infection maps closely to their sequential order on the T7 genome [15,16]. By relocating an essential early gene, encoding the T7 RNA polymerase to downstream positions, one delays initiation of transcription by the highly efficient T7 RNA polymerase and thereby attenuates phage growth [17]. Preliminary models for the growth of phage carrying the altered genomes, based on wild-type parameters, capture the overall observed trends in attenuation. Here we expand this approach to a mammalian virus of biomedical and agricultural relevance: vesicular stomatitis virus (VSV).

VSV is a prototype negative-sense single-stranded RNA (*Mononegavirales,* (-)ssRNA) virus and a member of the family Rhabdoviridae [18,19], which includes rabies virus. Each VSV particle has a single copy of an 11-kb genome carrying five genes that encode nucleocapsid (N), phospho (P), matrix (M), envelope (G), and polymerase (L) proteins. VSV is economically important because it can cause symptoms like those of foot-and-mouth disease in livestock [19]. It offers several advantages as a vaccine vector including low seropositivity in humans, a capacity to accommodate foreign genes up to 40% of its own genome size, and an established reverse genetics [18,20]. Recombinant forms of VSV carrying foreign virus genes that encode immunogenic proteins have been proposed as potential vaccines against HIV, influenza, and respiratory syncytial virus [21–25]. Less pathogenic but more immunogenic VSV-based vaccines against infection by VSV or other viruses are being sought.

Here we develop an in silico model of a VSV infection cycle, incorporating known regulatory interactions and mechanisms and relevant quantitative data from the literature of the past 40 years. These interactions and the corresponding equation formulations are described in detail in the model development section of Materials and Methods. Using the model, we first quantitatively analyze how the intracellular growth of wild-type VSV directs host biosynthetic resources toward VSV gene expression, synthesis of progeny genomes, and pathway switching from the synthesis of VSV intermediates to the production of VSV progeny. We then reveal that the model captures experimental results showing progressive attenuation of virus growth associated with moving the N gene downstream from its wild-type position. Finally, we use the model to predict how altering the positions of other VSV genes and promoters may attenuate the growth of VSV while increasing its potential capacity to activate an adaptive immune response.

## Results/Discussion

### Quantitative Features of VSV Regulatory Mechanisms

Using our model with the established parameter set (Tables 1 and 2), we first analyzed quantitatively and systematically how the intracellular growth of VSV is regulated. The improved understanding of the virus infection by this model-based analysis may guide us to identify the key regulatory components to manipulate for developing virus mutants as possible vaccine or vector candidates.

**Table 1:**
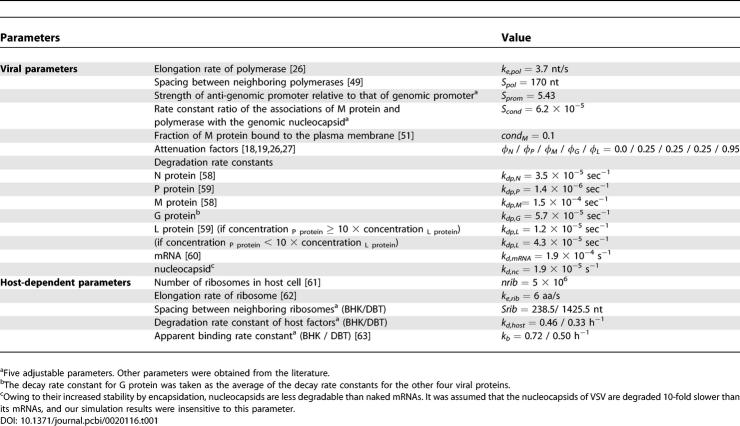
Model Parameters

**Table 2:**
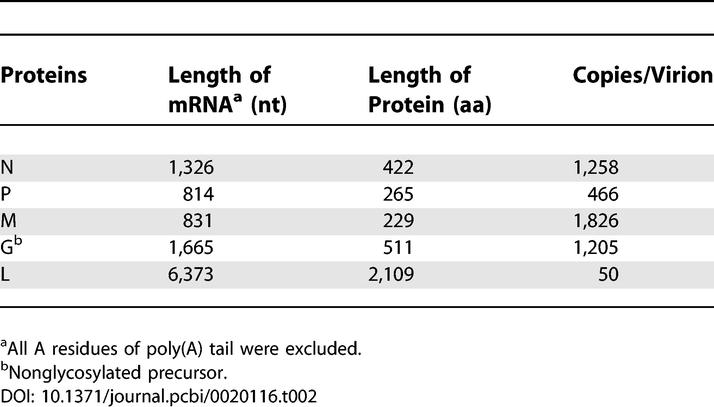
Protein Composition of VSV Particle and Lengths of Its Encoded Products

#### Attenuation mechanism leads to unequally distributed synthesis of viral mRNAs and proteins.

The partial transcription termination mechanism (or attenuation) is common in (-)ssRNA viruses. This mechanism is important to satisfy the different needs of each viral protein during its infection cycle. Five attenuation factors for each intergenic region of the VSV genome (Table 1 and Equation 8) were obtained from the literature [18,19,26,27] and incorporated into our model.

**Figure 1:**
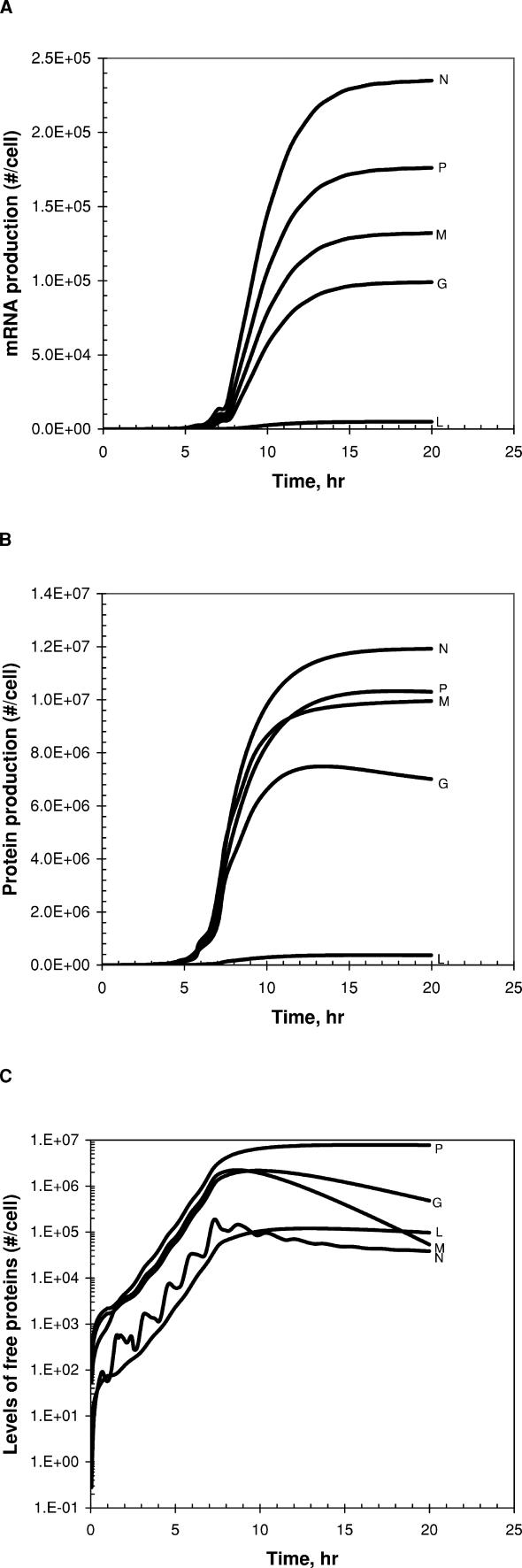
Estimated Production of Viral mRNAs and Proteins from Infected BHK Cells All the viral proteins that are in the cytoplasm, plasma membrane, or assembled virion particles are counted. (A) Viral mRNAs. (B) Viral proteins. (C) Estimated levels of free viral proteins in infected BHK cells. Free viral proteins include all the proteins in the cytoplasm or plasma membrane, but exclude the proteins that are incorporated into nucleocapsids and virion particles. V_ex_(0) = 3.

Owing to the step-wise release of polymerases from each gene junction, our simulations estimated the gradual decrease of VSV mRNA synthesis in the order of N > P > M > G > L (Figure 1A). Compared with the most abundant N mRNA, L mRNA is very scanty in infected baby hamster kidney (BHK) cells (40 ~ 140-fold less). The relative production level of each protein matched the relative availability of the corresponding mRNAs (Figure 1B) even though different proteins degrade at different rates (Table 1). Because of the different level of incorporation of each protein into a single virion particle, as defined by the protein stoichiometry (Table 2, [28,29]), the relative levels of free viral proteins in the cytoplasm develop differently from their mRNA levels (Figure 1C). P protein is most abundant owing to its low content in the virion, and L and N proteins are least abundant. The persistently low level of N protein is related to its immediate complexation with nascent genomes and anti-genomes to make nucleocapsid particles during the replication step. Owing to the cyclic switching between transcription and replication by the encapsidation process, the N protein level is predicted to oscillate as shown in Figure 1C. After 9 h post-infection, our simulation predicts a significant decrease of free M proteins. This arises from the dominance of the virion assembly process, which depletes M proteins, compared with transcription and replication. In infected murine delayed brain tumor (DBT) cells, similar distributions of viral mRNAs and proteins were obtained (unpublished data).

#### Higher demands for genomes are satisfied by the stronger promoter of the anti-genome template relative to that of the genome template.

**Figure 2:**
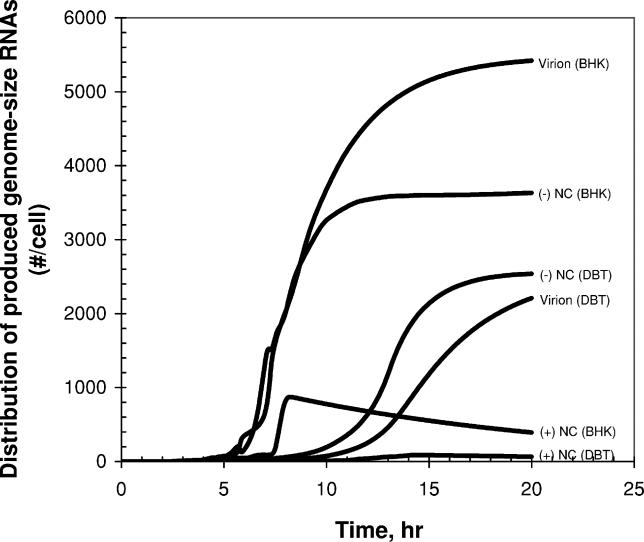
Estimated Distribution of Genome-Size Viral RNAs That Are Produced in Infected BHK and DBT Cells Virion and (-)NC indicate viral genomes that are incorporated into virion particles and intracellular nucleocapsids, respectively. (+)NC indicates anti-genomes that are incorporated into intracellular nucleocapsids. V_ex_(0) = 3.

Anti-genome templates are only utilized to amplify genomes, while genome templates are used to amplify both anti-genomes and mRNAs, and they are also incorporated into virion progeny particles. The higher demands for genome by these multiple tasks are satisfied by the stronger promoter of the anti-genome template compared with that of the genome template [30–32]. More polymerases bind to the stronger promoter of the anti-genome, ultimately enhancing the production of genomes over anti-genomes (Figure 2). In our model the parameter *S_prom_* measures the strength of the anti-genomic promoter relative to that of the genomic promoter (Table 1). The production ratio of genomes to anti-genomes was estimated to be dynamically changed [33], varying from one to 30 (wild-type VSV case (*S_prom_* = 5.4), Figure S1). Such oscillatory changes in the production ratio shown in Figure S1 follow from the on–off use of the genomes for transcription or replication. They also arise owing to the staggered shifting of dominant templates between genomes and anti-genomes during replication. A large value of *S_prom_* favors use of anti-genome templates to replicate genomes. However, as genome templates accumulate in large excess relative to anti-genome templates, they successfully compete for replication resources. Synthesis of anti-genomes then dominates until they accumulate and serve again as the dominant templates.

The virion production rate in BHK cells is at maximum 5–10 h post-infection. In infected DBT cells, similar simulation results were obtained except that the synthesis of genome-sized viral RNAs continued for longer time (active until 15 h post-infection, Figure 2).

#### Optimal utilization of genomic nucleocapsids.

Genomic nucleocapsids can either be used as templates for RNA synthesis or they may be incorporated into progeny virions. Their fate depends on levels of polymerase and M protein, which respectively favor RNA synthesis or virion production pathways, as well as on the extent to which association of the nucleocapsid with M protein will dominate over association with polymerase, described with the parameter *S_cond_* in our model (Table 1). Because both RNA synthesis and virion production are essential processes of the infection, extreme values of *S_cond_* that favor one process over the other will be detrimental for growth. For excessively large *S_cond_*, newly synthesized genomic nucleocapsids would tend to be prematurely incorporated into virion particles before they could serve as templates for transcription and replication. On the other hand, for extremely small *S_cond_,* genomic nucleocapsids would be utilized primarily to produce viral RNA without being packaged into viral progeny. Hence, an intermediate parameter value is expected to be optimal for viral growth. We estimated a possible range for the wild-type value of *S_cond_* by fitting our simulation results to previous experimental observations by others (2.5 × 10^−5^ to 1.0 × 10^−4^, Figure S4). Our simulations further indicate that this range of *S_cond_* is near-optimal and optimal for VSV growth on BHK and DBT cells, respectively (unpublished data).

#### Diversion and inhibition of host translation machinery create a time window of opportunity for translation of viral proteins.

**Figure 3:**
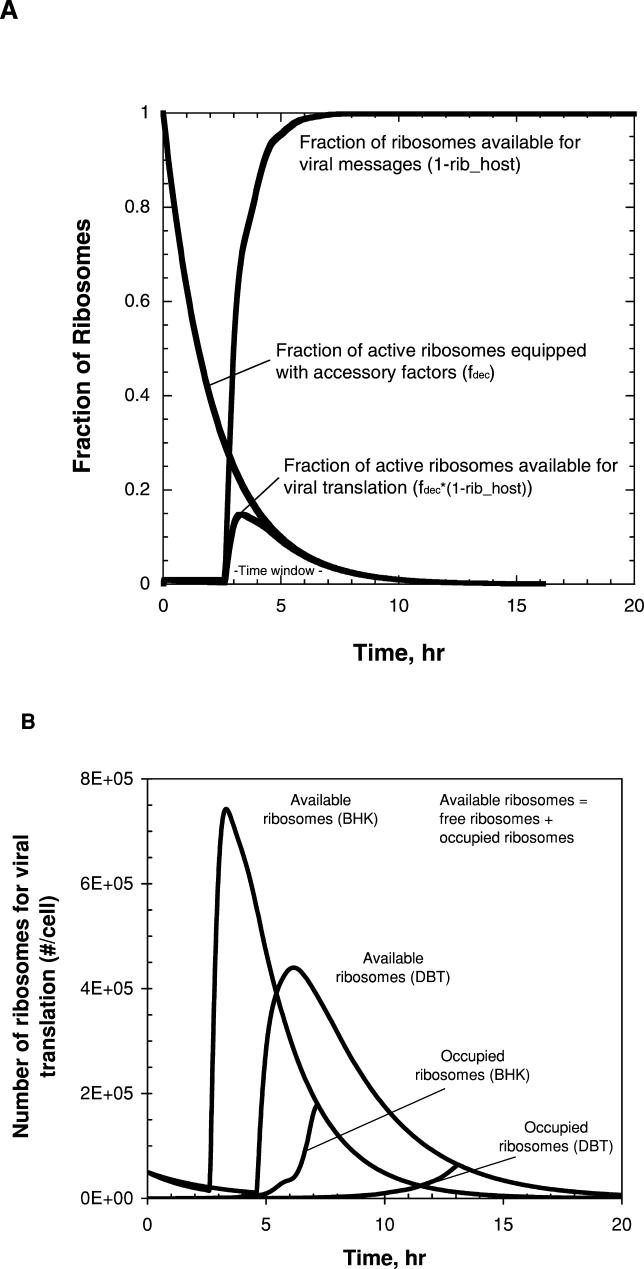
Hijacking of Host Translation Resources by Virus (A) The estimated distributions between active (equipped with the required accessory factors) and inactive (not equipped) ribosomes, and between ribosomes available for viral and host mRNAs are shown. “- Time window -” indicates the time period during which viral translation is actively supported by host ribosomes. (B) The estimated numbers of ribosomes available for viral translation and actually occupied by viral mRNAs are shown for the cases of infected BHK and DBT cells. Non-occupied ribosomes are considered as free ribosomes. When two lines coincide, no free ribosomes exist (all the available ribosomes are occupied by viral mRNAs). V_ex_(0) = 3.

During the infection cycle, virus actively and passively competes with the host for limited translation resources by inhibiting host transcription and by amplifying viral mRNAs, respectively. Viral leader–mRNA and M protein play key roles in this inhibition [19,28,34–36]. As viral components accumulate in the cytoplasm from the initiation of infection, an ever-increasing fraction of host ribosomes are available for viral mRNAs (Figure 3A, the fraction of ribosomes associated with viral mRNAs is defined by 1- *rib_host* in Equation 26). However, the inhibition of host macromolecular synthesis causes a failure to supply accessory factors needed for initiation and elongation steps of translation, resulting in a reduction in the fraction of active ribosomes over time (Figure 3A, as described by *f_dec_* in Equation 25). These two mechanisms create a time window when active ribosomes are maximally available for viral translation in infected cells (Figure 3A, refer to the term (1- *rib_host*)*f_dec_ in Equation 27). The abundance of viral mRNAs and the limitation imposed by ribosomal spacing determine the fraction of the active ribosomes involved in translating viral mRNAs (occupied ribosomes in Figure 3B, refer to Equations 11 and 27). In our model, if the occupied active ribosomes are less than the available ones (in this case the number of free active ribosomes > 0), viral translation is fully supported without any limitation of host machinery. In the early infection stages up to 7 h and 13 h post-infection for BHK and DBT cells, respectively, the host machinery is in excess (Figure 3B). However, at later times viral translation becomes limited by the host resources (in this case the number of free active ribosomes = 0). This limitation may cause a transition from replication-dominant to assembly-dominant infection stages because the replication requires the continuous protein synthesis. As shown in Figure 3B, a small fraction of ribosomes as active forms (less than 5% out of 5 × 10^6^ ribosomes, Table 1) are utilized for viral translation.

#### Experiments and simulations of VSV gene-order mutants.

For vaccine applications, one seeks to minimize viral pathogenicity and maximize its immunogenicity. Based on observed correlations between in vitro and in vivo results, we assume here that the pathogenicity and the immunogenicity of a virus are directly linked to the levels of progeny production [20,37,38] and G protein expression in infected cells [39,40], respectively. In the previous section we have showed that various VSV regulatory mechanisms are involved in maintaining balances, during infection, among viral synthesis processes, which indirectly indicates the importance of such balances for viral growth. Perturbations of such balances by genetic or genomic manipulations could provide ways to obtain viral phenotypes favorable to vaccine applications. We first test the predictive ability of our model by comparing simulated protein expression and growth of several gene-rearranged VSV strains with experimental results. Later we employ the model to predict how various genomic manipulations could attenuate virus growth and increase G protein expression.

#### Protein expression rates of gene-rearranged viruses.

**Figure 4:**
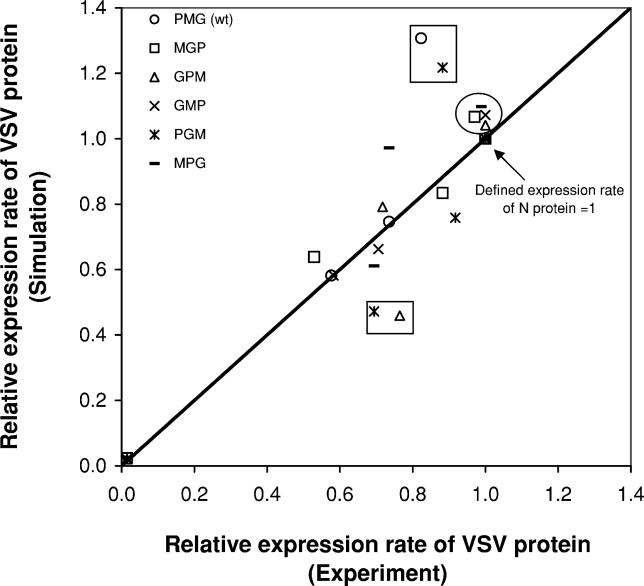
Relative Expression Rates of Viral Proteins in BHK Cells Infected by Individual VSV Strain The expression rate of each protein was normalized by that of N protein. Therefore, the relative expression rate of N protein is defined as one. X and Y coordinates of datapoints indicate the results of experiments and simulations, respectively. All the experimental datapoints were obtained from the literature [18]. The four datapoints in the circle denote the relative protein expression rates for the genes located at the second genome position in the cases of MGP, MPG, GPM, and GMP strains having gene orders 3′-N-M-G-P-L-5′, 3′-N-M-P-G-L-5′, 3′-N-G-P-M-L-5′, and 3′-N-G-M-P-L-5′, respectively. PMG and PGM strains have the gene orders 3′-N-P-M-G-L-5′ and 3′-N-P-G-M-L-5′, respectively. The two rectangular boxes include four datapoints where there are the largest discrepancies between simulation and experimental results. V_ex_(0) = 5.

The stepwise decline in the transcription of genes more distant from the 3′-end region promoter highlights how gene order affects gene expression in VSV. Advances in reverse genetics have made it possible to create gene-rearranged virus strains where the transcriptional attenuation mechanism then creates altered levels of gene expression [7,9,18,20]. In one study [18] the three internal genes, P, M, and G, were permuted, and the resulting six possible VSV strains were characterized. Relative rates of viral protein expression in BHK cells were experimentally measured based on their incorporation of [^35^S]-labeled methionine for a one-hour window at 4 h post-infection [18]. We extended our model to simulate this experiment for mutants representing each gene-order permutation and compared the model prediction with the published results [18], as shown in Figure 4. All rates are expressed relative to the synthesis rate of N protein, whose corresponding gene was in position 1, closest to the 3′ end of the genome in all strain cases. Expression of gene L, in position 5, was minimal in both simulations and experiments, and the expression of all other genes was above 40%, a feature of the experimental data that the simulation also captured. Most of the points fall close to the parity line, indicating agreement between the simulation and experiment. Noteworthy are two subsets of points. First, the four circled points are exceptions to the general rule that gene order determines the level of gene expression. These were genes in the second position of the genomes that were expressed essentially at the same rate as gene N, in the first position [18]. This result highlights that the expression rate of protein is affected not only by its gene order, or corresponding rate of mRNA production, but also by its length and degradation rate. For a fixed average rate of translational elongation, longer gene products will tend to be produced more slowly. Further, the net rate of protein production will reflect the rates of both protein synthesis and protein degradation. The model accounts for these contributions, and for the circled genes such accounting appears to capture unexpected high translation levels of genes in the second position, which were measured by Ball et al. [18]. The second sets of points, shown in two boxes, indicate mismatches (Figure 4) that, in the most challenging scenario, could reflect unknown strain-specific mechanisms that are not present in our general gene-permutation model. However, one should also note that the experiment is based on labeling and quantifying proteins about 4 h post-infection. This relatively early time point allows one to minimize potentially confounding influences of virion particle assembly and production on cytoplasmic levels of viral proteins, but it also represents a point before the majority of viral proteins have been made (Figure 1B).

#### Growth of gene-rearranged viruses.

**Figure 5:**
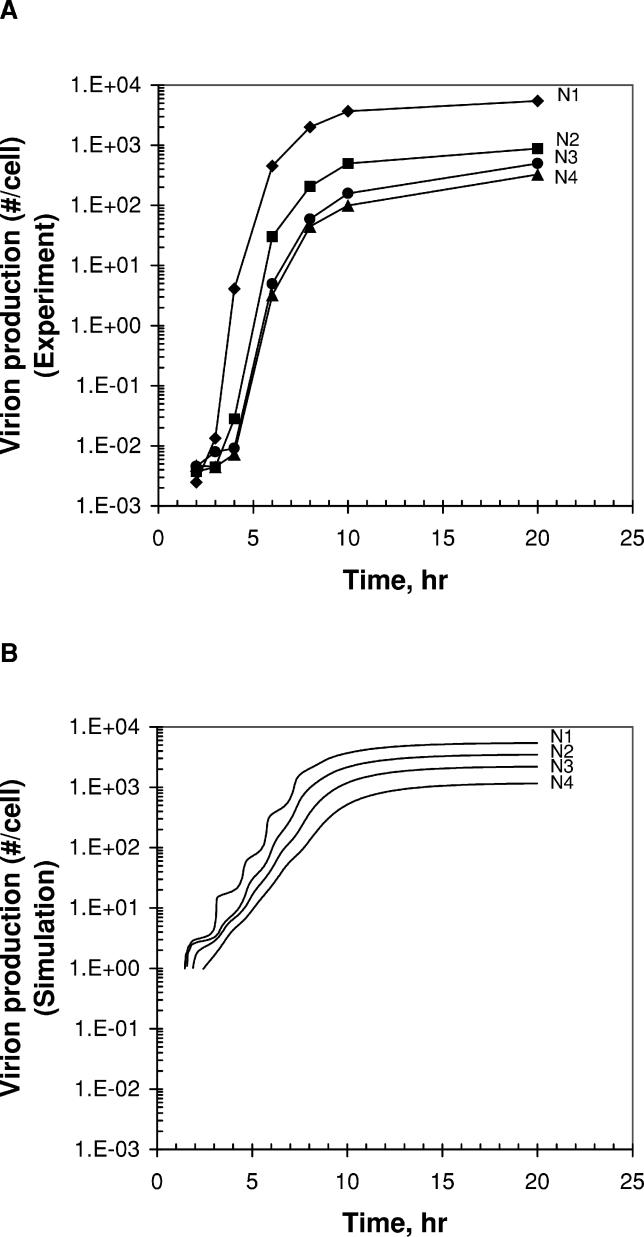
The Growth of Gene-Rearranged VSV Strains in BHK Cells (A) Experimental data. (B) Simulation results. The growth of the N1 VSV strain is the fitting result, but the growth of the other three strains is the model prediction result. V_ex_(0) = 3.

We also employed our model to predict the growth of VSV strains having the N gene at four different locations (N1 [wild-type], N2, N3, and N4) and then compared the simulation results with the experimental data. N1, N2, N3, and N4 VSV strains have the gene orders, 3′-N-P-M-G-L-5′ (N1), 3′-P-N-M-G-L-5′ (N2), 3′-P-M-N-G-L-5′ (N3), 3′-P-M-G-N-L-5′ (N4), respectively. As shown in Figure 5, our simulations qualitatively matched the experimentally observed relative growth of the four strains in BHK cells. The wild-type virus having the N gene at the first position on the genome grows best, followed by N2, N3, and N4. This result is consistent with the previously suggested hypothesis that relocation of the N gene to 3′-distal positions on the genome would be an efficient way to attenuate VSV for vaccine use [20]. The reduction in growth that follows from moving the N gene likely reflects, at least in part, an imbalance between replication and transcription. Insufficient production of N protein would reduce the extent of encapsidation of nascent anti-genome and thereby allow transcription to dominate over genome replication [41]. While the simulation matches the growth ranking, it did not quantitatively match the experimental data. The predicted variation in virion production (N1 → N4: 4.7-fold decrease) is smaller than the experimentally observed variation (N1 → N4: 16.7-fold decrease). A potential source of this quantitative difference was our neglect of mass action effects of N proteins on the encapsidation process in our model; encapsidation was simulated as an instantaneous process when free N proteins were available. As shown above, our model could capture the major effects of gene rearrangement on viral growth and protein expression.

#### Effects of relative promoter strength on viral growth.

**Figure 6:**
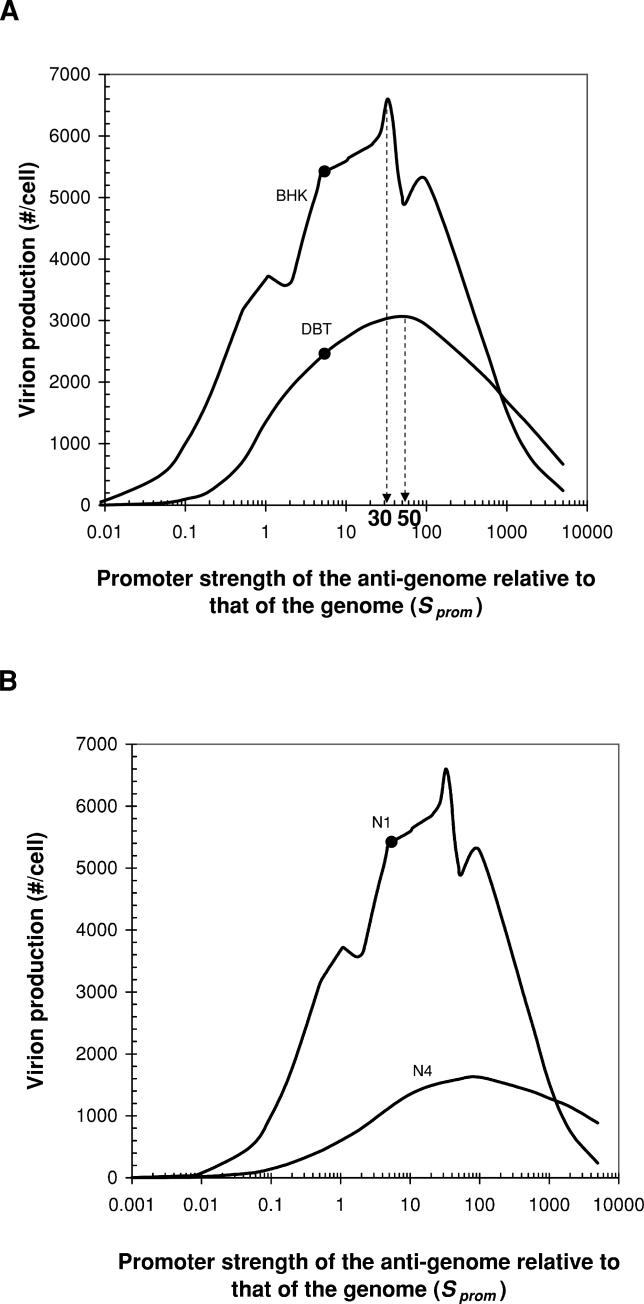
The Effects of Relative Promoter Strength and N Gene Rearrangement on the Growth Phenotype of VSV (A) The balance of the production between genome and anti-genome is determined by the promoter strength of the anti-genome relative to that of the genome (*S_prom_*). The changes of virion production in BHK and DBT cells by the variation of the parameter are observed. The black circles indicate the virion productions of wild-type (*S_prom_* = 5.4) in BHK and DBT cells. (B) The extension of phenotypic variations by double genomic manipulations was predicted. As an important phenotype of virus for vaccine use, the changes of virion production by the relocation of N gene along with the variation in the promoter strength of the anti-genome relative to that of the genome (*S_prom_*) are shown. The black circle indicates the virion production of wild-type in BHK cells. V_ex_(0) = 3.

The genome and anti-genome of VSV are synthesized in unequal amounts, determined by the differing strengths of their promoters [19,31,33,39]. The stronger promoter of the anti-genome allows viral polymerases to produce more genomes to meet their demands as components of virion particles and as templates for transcription and replication. To explore how VSV growth is influenced by differences in the relative strength of the genomic and anti-genomic promoters, we predicted the yield of virus on BHK and DBT cells over a broad range of *S_prom_*, as shown in Figure 6A. Small *S_prom_* virus cannot grow well because most polymerases would be associated with genomes and tend to synthesize primarily anti-genomes. For example, for *S_prom_* equal to 0.1, infected BHK and DBT cells make 5-fold and 26-fold fewer progeny than wild-type VSV infected cells, respectively. However, large *S_prom_* virus also cannot grow well because most polymerases would preferentially bind to newly synthesized anti-genomes, producing few of the anti-genomes that are needed as templates to amplify genomes. Our simulations predicted that values of 30 and 50 for *S_prom_* would be optimal for VSV growth in BHK and DBT cells, respectively (Figure 6A). The estimated wild-type value of *S_prom_* of 5.4 gives VSV yields higher than 80% of their maximum yields for both cell types (Figure 6A).

We speculate that a rational way to attenuate the pathogenicity of the live wild-type virus would be to swap its two promoters, giving an *S_prom_* of (5.4)^−1^. For this promoter swap we predict virion production would be decreased by 3.3-fold and 14.5-fold for infected BHK and DBT cells, respectively, relative to wild-type. However, the extent of growth attenuation by the promoter swapping can be higher than the model prediction because the swapping may also perturb viral transcription and virion assembly and budding processes modulated by the signals encoded in the 3′and 5′ termini of the genome [42,43] that are not yet sufficiently defined to be included in the simulation.

#### Rational vaccine attenuation by double genomic manipulations.

Several variant VSV strains, including N1 through N4, have been made by Ball and Wertz [18,20]. With an aim to generate a potentially broader diversity of growth phenotypes, we created and tested in silico VSV mutants by combining N gene relocations with a range of *S_prom_*. This is a computationally simple task, but experimentally nontrivial. For example, the VSV N4 with *S_prom_* = 0.1 produced a simulated 38-fold fewer virus progeny than wild-type in BHK cells (Figure 6B). It is interesting to note that this 38-fold degree of growth attenuation is greater than the product of the constituent attenuations (4.7 × 5.4 = 25) that one calculates by assuming that the effects were uncoupled. Such nonmultiplicative effects of double genomic manipulations on growth would be challenging to predict in the absence of a quantitative model.

#### Modulation of VSV immunogenicity by gene shuffling.

**Figure 7:**
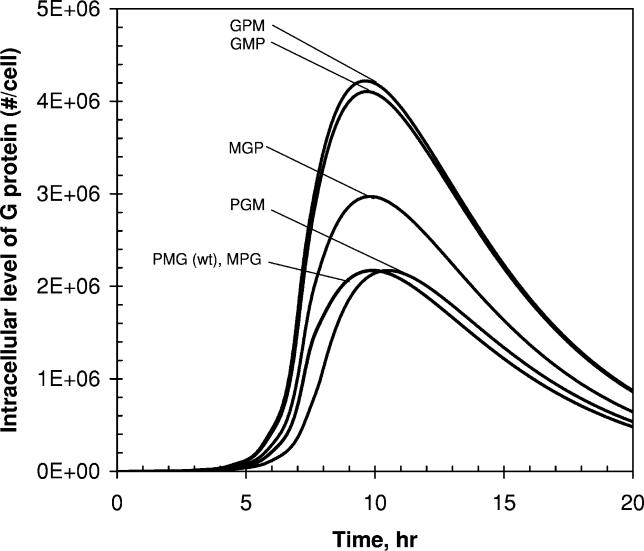
Estimated Levels of G Protein in BHK Cells Infected with Six Gene-Shuffled VSV Strains The intracellular level of G protein is highly dependent upon the location of G gene on the viral genome. In the late infection stage, a large fraction of G proteins are incorporated into progeny particles, which significantly decreases the intracellular protein level. V_ex_(0) = 3.

To elicit a systematic immune response, live viral vaccines must present or display neutralizing epitopes, typically through the expression of viral surface proteins. Higher levels of antigen expression have been found to correlate with more rapid and potent induction of anti-viral antibodies [39,40]. As Flanagan et al. suggested, the gene encoding G may be moved to other positions in the VSV genome to modulate the expression of the VSV surface glycoprotein G [39]. We generated in silico five gene-shuffled VSV strains, having gene orders for the three internal genes, MPG, MGP, PGM, GMP, and GPM, and simulated levels of G protein in BHK cells infected with those strains. Our simulation results were consistent with the idea that the location of G gene affects the production of G protein. The GPM strain gave the highest concentration of G protein in the cytoplasm (almost 2-fold higher than that of wild-type, Figure 7). However, in many cases effects of such gene rearrangements can be difficult to anticipate because of the complexity of the involved interactions among viral components. For example, the PGM strain showed only a level of G protein expression similar to those of PMG (wt) and MPG strains even though it has the G gene at an earlier position than the other two strains (Figure 7).

For vaccine use we might aim to maximize the immunogenicity of VSV or a VSV-based vector through the expression increase of VSV G gene or inserted foreign gene while minimizing their potential pathogenicity by growth attenuation. Given such design goals, specifically for a VSV vaccine, we might prefer strain GPM, which showed the highest expression of G protein (Figure 7) and the lowest production of virions [18]. Toward such favorable features, Flanagan et al. previously constructed three VSV strains having the following gene orders: 3′-G-N-P-M-L-5′ (G1N2), 3′P-M-G-N-L-5′ (G3N4), and 3′-G-P-M-N-L-5′ (G1N4) [39]. These genome constructions were based on their intuitive idea that translocations of G gene and N gene to earlier and later positions, respectively, compared with wild-type, could not only increase the expression of G protein, but also attenuate virus growth [39]. This idea was supported by their experimental results [39].

**Figure 8:**
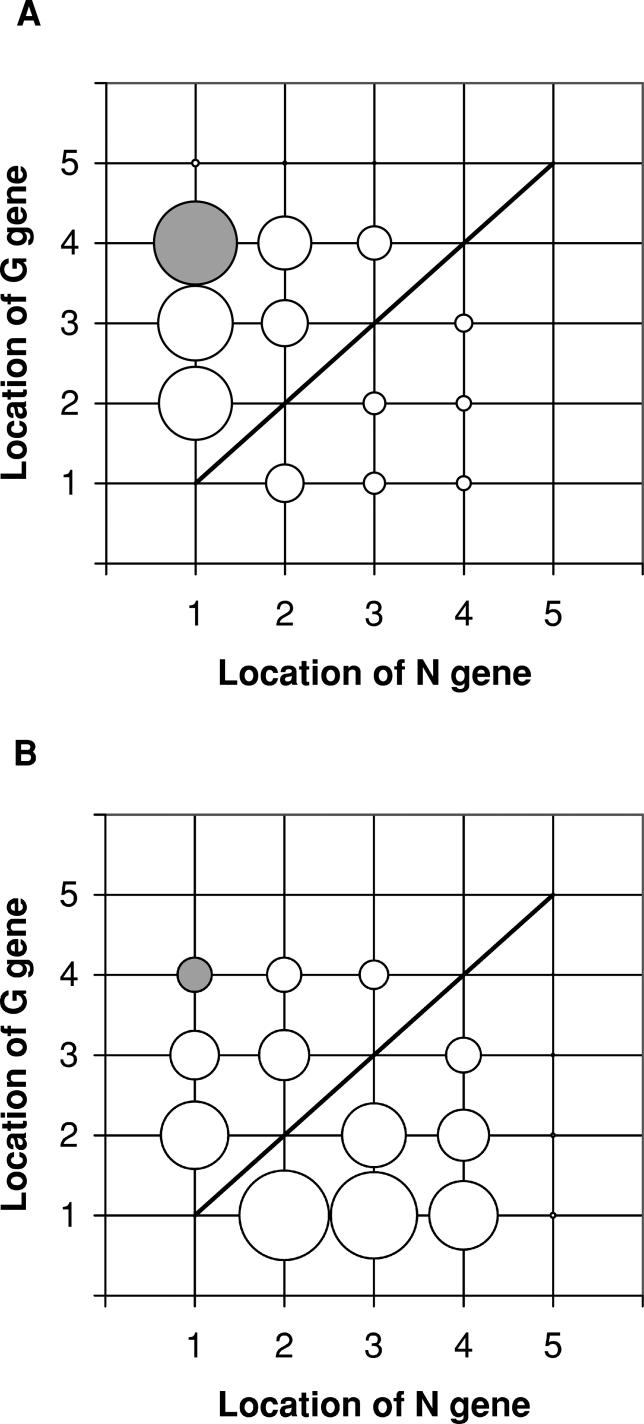
Predicted Levels of Virion Production and G Protein Expression in BHK Cells Infected with VSV Strains (Total 20 Strains) (A) Virion production. (B) G protein expression. The size of each circle shows the relative level for each VSV strain, and the coordinate of each circle indicates the gene order in each strain's genome. The gray and white circles denote the cases of wild-type (N1G4) and non–wild-type strains, respectively. Under the line there is no VSV strain case (e.g., N1G1, N2G2, etc.). V_ex_(0) = 3.

Seeking a more detailed correlation between locations of the two genes and the viral phenotypes relevant to vaccine application, we simulated in silico the growth of all mutants that retain the gene order P – M – L of the wild-type, but allow G and N to move, criteria that define 20 possible gene-order permutations. The viral growth and the level of G protein in infected BHK cells mainly depend on the locations of N gene and G gene, respectively (Figure 8A and 8B), which is consistent with the experimental results of Flanagan et al. [39]. Further, if gene G is fixed, then moving gene N closer to the 3′ promoter is predicted to increase protein G expression (Figure 8B). Consistent with this prediction, Flanagan et al. also observed a higher G protein expression for the G1N2 strain than for the G1N4 strain [39]. Enhanced replicative ability of VSV strain by locating its N gene at an earlier position in its genome can contribute to increasing the level of G protein. If either gene N or gene G is located at the fifth position, then both levels of virus growth and G protein expression are very low (Figure 8), because with such genome organizations the stoichiometric amounts of N and G proteins required for replication and assembly (Table 2, [28,29]) cannot be reached. Our simulations with the BHK cell parameters (Table 1) overall captured the experimentally established relative growth of the VSV strains in BHK-21 cells, but the growth of the G3N4 strain was significantly overestimated compared with the experimental results [39] (Table 3). The relevant mechanism for such a large discrepancy between the simulation and the experimental results remains to be elucidated.

**Table 3:**
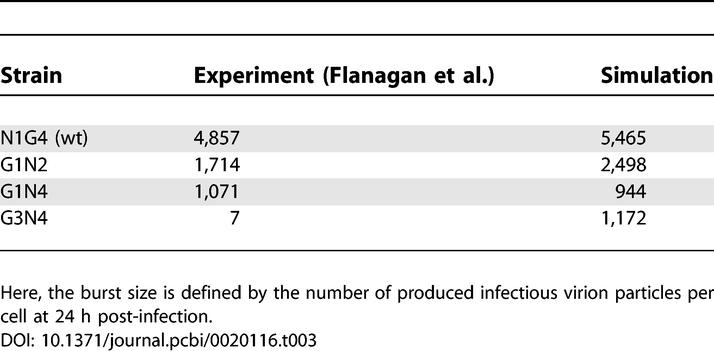
Burst Size of VSV Strains

The changes of protein expression levels by gene shuffling can be a rational means to modify the viral features for vaccine use. Robust synthesis of antigen by a highly attenuated strain appears to be an effective vaccine strategy as Flanagan et al. previously suggested. In addition to controlled attenuation of virus growth, a potent vaccine should ideally elicit a strong humoral or cell-mediated immune response.

### Perspectives

In the era of highly advanced genetic technologies, we have witnessed a turning point for the development of live viral vaccines. Conventional empirical vaccine development processes are now being replaced by more rational reverse-genetics–based ones. With this trend, much attention will be focused on mechanism-based design of less pathogenic and more immunogenic virus stains. Mathematical models for intracellular virus growth can support this design process by providing a tool to systematically analyze the viral infection regulatory network, identify critical regulatory mechanisms or components for redirecting viral phenotypes, and reverse engineer desirable phenotypes.

## Materials and Methods

### Experiments: Cell and virus culture.

BHK cells were obtained from I. Novella (Medical College of Ohio, Toledo, Ohio, United States) and grown as monolayers at 37 °C in a humidified atmosphere containing 5% CO_2_. BHK growth medium was Minimal Essential Medium with Earle's salts (MEM) (Cellgro, Fisher Scientific, http://www.fischersci.com) containing 10% fetal bovine serum (FBS, HyClone, http://www.hyclone.com), and 2 mM Glutamax I (Glu, Gibco, http://www.invitrogen.com). DBT cells were obtained from J. Fleming (University of Wisconsin-Madison, Madison, Wisconsin, United States) and grown under the same environment as the BHK cells. DBT growth medium was Dulbecco's Modified Eagle Medium (Cellgro, Fisher Scientific) containing 10% newborn calf serum (NCS, Hyclone), 4 mM Glutamax I (Glu, Gibco), and 15 μM HEPES (Sigma, http://www.sigmaaldrich.com). Both BHK and DBT cells were subcultured approximately every third day. For subculture, cell monolayers were rinsed with Hanks Balanced Salt Solution (HBSS, Fisher Scientific), incubated in 0.025% trypsin/26 mM EDTA (Fisher Scientific) for 5 min, dispersed through mixing, and then replated in fresh growth medium at 1:30 (BHK) or 1:15 dilution (DBT). No antibiotics were used, and cells were subcultured for no more than 4 mo to minimize the effects of cell senescence. Viability of cell populations always approached 100%, as determined by trypan blue exclusion at the time of experiments. Four gene-rearranged strains (N1–N4) of VSV were obtained from G. Wertz (University of Alabama School of Medicine, Birmingham, Alabama). Their genomic structures are as follows: 3′-N-P-M-G-L-5′ (N1), 3′-P-N-M-G-L-5′ (N2), 3′-P-M-N-G-L-5′ (N3), 3′-P-M-G-N-L-5′ (N4). Each strain of virus was bulked once on BHK cells at multiplicity of infection (MOI) of 0.01 or 0.1 plaque-forming units per cell. Infected cells were incubated in infection medium (MEM/Glu/2% FBS) for 20–24 h. At the end of the incubation period, the medium was harvested and passed through a 0.2-μm filter. The filtered virus solution was aliquoted and stored at −90 °C until use.

### One-step infection of cell monolayers.

Cells were harvested, resuspended in growth medium, and plated into six-well plates at a concentration of 5 × 10^5^ cells per 2 ml per well. Plated cells were returned to the incubator and allowed to grow overnight. The next day, two representative cell monolayers were harvested and counted to give an approximate number of cells per well. Each monolayer was then incubated with 200 μl of virus inoculum (MOI 3) for 1 h to allow virus adsorption. The plates were rocked gently every 20 min to evenly distribute virions on the monolayers during the adsorption step. After the adsorption period, the monolayers were rinsed twice with 1 ml of HBSS and then placed under 2 ml of infection medium for incubation. Medium samples of 200 μl including virion particles were taken from each well at 2, 3, 4, 6, 8, 10, and 20 h post-inoculation. Samples were kept frozen at −90 °C until their analysis by the plaque assay.

### Plaque assay.

BHK cells were plated into six-well plates and cultured to 90% confluence. Culture medium was removed from each well and replaced with 200 μl of serially diluted viral samples. The inoculated monolayers were returned to the incubator for 1 h to allow virus adsorption. The plates were rocked gently every 20 min. At the end of the adsorption period, the inoculum was removed from each monolayer sample and then replaced with 2 ml of agar overlay. The agar overlay consisted of 0.6% weight/volume (w/v) agar (Agar Nobel, Difco, BD Diagnostic Systems, http://www.bd.com). 5-Bromo-2′-deoxyuridine (B5002, Sigma) was added, at 100 μg/ml, to the agar overlay of N3- and N4-infected samples to enhance plaque formation. Following agar addition, the plates were allowed to cool at room temperature for 30 min, returned to the incubator and incubated for 24 h, and then each sample was fixed with 2 ml of fixative for 3 h at room temperature. The fixative consisted of 4% (w/v) paraformaldehyde (VWR) and 5% (w/v) sucrose (Sigma) in 10 mM phosphate buffered saline (PBS, Sigma) of pH 7.4. The agar overlay was then removed, and each sample was rinsed twice with 2 ml of PBS. Gentian violet diluted in methanol (0.01% (w/v), Sigma) was used, at 1 ml each, to stain the samples.

### Model development.

Using algebraic and differential forms of equations, our mathematical model aims to account for established molecular processing steps in the development of VSV. Most model parameters were extracted from the literature. However, five parameters were obtained by fitting our simulation results to experimental data that were from the literature and our own experiments. Key model parameters are given in Table 1, and detailed descriptions of the model and parameter estimation process are provided below, and in Protocol S1 and Figures S2 and S3, respectively.

### Virus binding and penetration.

**Figure 9:**
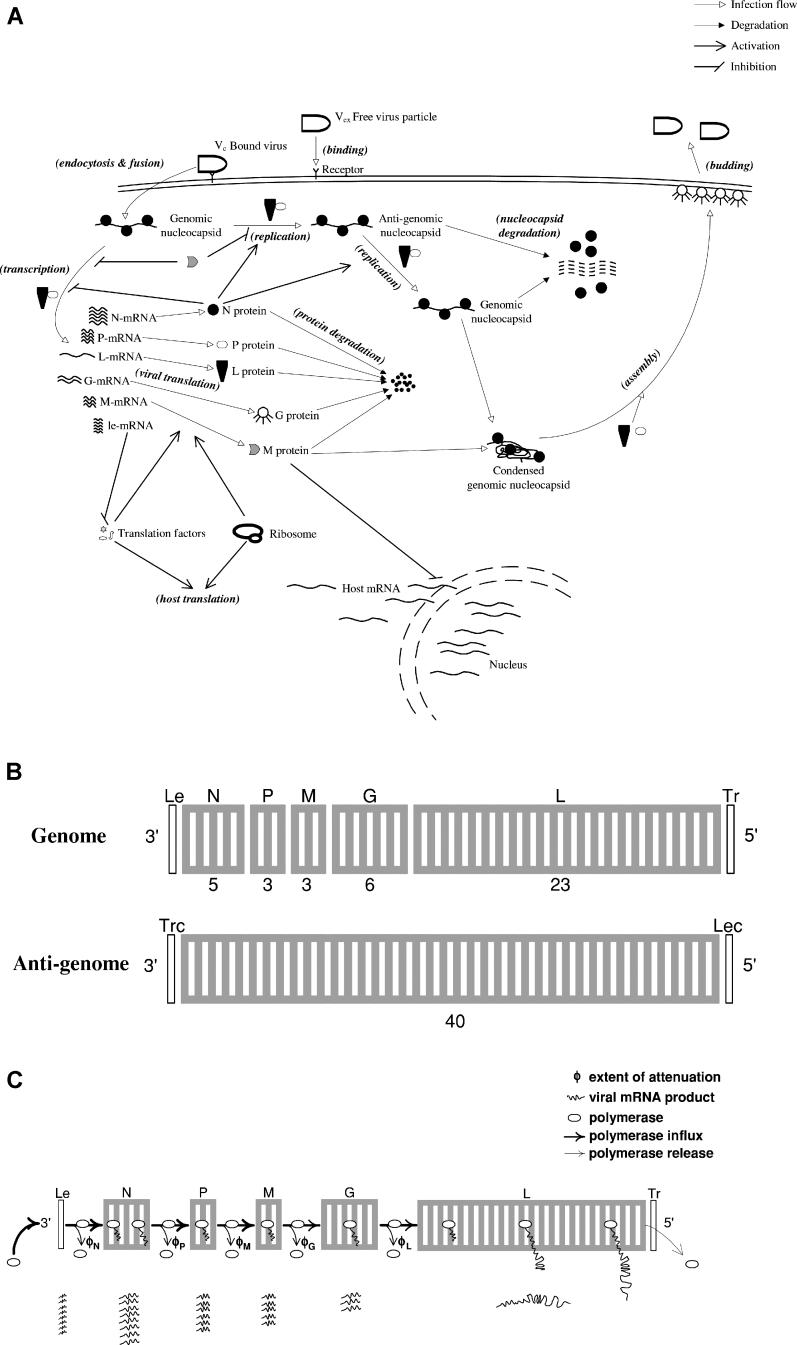
Schematic Descriptions (A) Infection cycle of VSV. (B) Segmentation of genome-size viral templates to simulate the spatial–temporal changes of polymerase concentration on the templates. (C) VSV partial transcription termination (or attenuation) mechanism.

As shown in Figure 9A, VSV initiates an infection by binding to a receptor such as phosphatidyl serine, a lipid component in the plasma membrane [19,44]. After the binding step, the VSV particle is endocytosed via a clathrin-coated pit, and then penetrates intracellular vesicles such as endosomes by membrane fusion [19,45–47]. The penetration leads to the release of the encapsidated negative-sense viral genome and virus proteins into the cytoplasm of the host cell. By assuming the binding step is rate-determining [48], we lump these early steps from the binding to the penetration into a first-order expression:

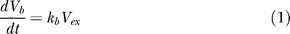
where *V_b_* and *V_ex_* are the concentrations of bound and extracellular virus particles, respectively, *t* is time, and *k_b_* is the apparent rate constant for virus binding. After binding, we assume the bound virus is immediately endocytosed and fused, and its genome and protein components are instantaneously released into the cytoplasm at the expense of the fused virus particle. The protein stoichiometry of a single VSV particle and the lengths of each viral gene and protein are summarized in Table 2, [28,29].

### Population distribution of polymerases and nucleocapsids.

Following the release of the encapsidated genome and proteins into the cytoplasm, VSV transcription is initiated. The viral transcription was assumed to be independent of host–cell functions such as replication [33]. Instead, the viral complex of L and P proteins, with a stoichiometry of 1–3.6, was taken to function as polymerase in transcription and replication [49]. In the absence of P protein, L protein cannot bind to the genome or anti-genome [50]. After binding to the 3′ promoter regions of the genomic and anti-genomic templates, the viral polymerase starts to synthesize its own RNA transcription and replication products by elongating along the templates. During transcription a fraction of elongating polymerases terminate transcription by dissociating from the templates as they encounter regulatory signals at intergenic regions [19,26]. In addition to the regulated polymerase dissociation, time-dependent concentration changes of the polymerases and the viral templates in the cytoplasm influence the distribution of polymerases on the viral templates during transcription and replication. Hence, the distribution of polymerases continuously varies over the viral templates, ultimately determining the relative synthesis levels of mRNAs and genome-size RNAs.

We simulate the transcription and replication processes by considering the spatial–temporal distribution of template-associated polymerases. We first partition the viral genome and anti-genome templates into 40 segments, excluding their 3′ and 5′ end regions, which are the leader (*Le*) and trailer regions (*Tr*) for the genome, and the complementary trailer (*Trc*) and complementary leader regions (*Lec*) for the anti-genome, respectively (Figure 9B). For the genome template that is used for transcription as well as for replication, we specially grouped the segments into five genes (Figure 9B). We chose 40 as a minimum number for total segments which allows each gene to be split into a specific integer number of segments, proportional to the length of the gene. By considering the mechanisms for the interactions between polymerase and the intergenic regulatory sequences of the templates, as described below in the Transcription section, we simulated the polymerase flux into each segment over the time elapsed from the initiation of transcription on each template. Then we correlated the level of polymerase occupying each gene-encoding section of the template with the synthesis rate of each corresponding viral mRNA. In a similar way, the distribution of polymerases on the replication templates was correlated with the synthesis rate of viral genome-sized RNA. Such explicit treatment of polymerase spatial distributions on the viral genome and anti-genome templates was central to modeling the growth of wild-type and gene-rearranged virus strains. This treatment systematically accounts for polymerization-associated time delays and the polymerase fluxes into each template segment.

Before estimating the polymerase flux, we need to figure out how the polymerase complex and M protein compete with each other for binding to the genomic nucleocapsids as well as how the polymerases bound to nucleocapsids are subsequently distributed to one of three possible tasks: transcription, replication of genome, or replication of anti-genome. In our model we assume that the genomic templates (negative-sense nucleocapsids) whose promoters (leader regions) are free of polymerases are available for association with free polymerase or M protein. We further assume that the associations of the free genomic templates by M proteins or polymerases take place instantaneously:

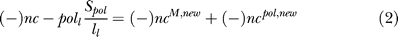
where (*-*)*nc*, (*-*)*nc^M,new^*, and (*-*)*nc^pol,new^* are the concentrations of total genomic nucleocapsids and subsets of genomic nucleocapsids whose promoters are newly occupied by M protein and polymerase, respectively. *S_pol_* is the spacing between neighboring polymerases on the genomic or anti-genomic template, *pol_l_* is the concentration of polymerases bound to the promoter region (*Le*) of the genomic template, and *l_l_* is the length of the promoter region. Specifically, the second term in the left-hand side of the equation denotes the concentration of the genomic templates whose promoters are currently occupied by polymerases. In our model the concentration of the genomic nucleocapsids whose promoters are bound to polymerases and the concentration of the polymerases bound to the promoters of the genomic nucleocapsids are interchangeable with each other by the factors (*l_l_*/*S_pol_*) and (*S_pol_*/*l_l_*), respectively. The binding of M protein or polymerase initiates reactions leading to virion assembly or RNA synthesis, respectively. Because the initiation of RNA synthesis by the polymerase requires a finite time, a space between adjacent polymerases on the template (*S_pol_*) would be maintained during infection, assuming a fixed elongation rate. With these considerations, one may expect that at any time the concentration of nucleocapsids available for the new binding of the free proteins is inversely proportional to the concentration of polymerases currently bound to the leader region of the genomic nucleocapsids (*pol_l_*) and the polymerase spacing (*S*_*pol*_) as shown in the second term of Equation 2. The ratio of (*-*)*nc^M,new^* to (*-*)*nc^pol,new^* is determined by the ratio of the association rates of M protein and polymerase with the genomic nucleocapsid, which is further a function of the rate constants and relative amounts of the corresponding free components in the cytosol:

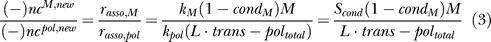
where *r_asso,M_* and *r_asso,pol_* are the rates of the associations of M protein and polymerase with the genomic nucleocapsid, respectively, and *k_M_* and *k_pol_* are the rate constants for each association reaction, respectively. *S_cond_* denotes the ratio of the two rate constants (=*k_M_*/*k_pol_*). Unlike L protein, 10% of synthesized M proteins are associated with the plasma membrane [51]. In Equation 3, *cond_M_* is the fraction of M proteins associated with the plasma membrane, *trans* is the fraction of L proteins satisfying the polymerase stoichiometry with P protein, *pol_total_* is the total concentration of polymerases associated at the time with nucleocapsids, and *M* and *L* are the total concentrations of M and L proteins not assembled into viral progeny. If the concentration of P protein (*P*) is larger than 3.6-fold concentration of L protein, then *trans* is equal to 1. Otherwise, *trans* is equal to *P*/(3.6*L*). In our model, M and L proteins compete for free genomic nucleocapsids, and the condensed nucleocapsids, owing to their association with M proteins, cannot be utilized for transcription or replication [19]. From Equations 2 and 3, the newly occupied nucleocapsids by polymerases ((*-*)*nc^pol,new^*) can be calculated:

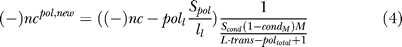
In the same way, given *S_pol_,* the concentration of positive-sense anti-genomic nucleocapsids available for binding to polymerases would be ((*+*)*nc – pol_trc_*(*S_pol_/l_trc_*)), where (*+*)*nc* is the total concentration of anti-genomic nucleocapsids, *pol_trc_* is the concentration of the polymerases bound to the promoter region (*Trc*) of the anti-genomes, and *l_trc_* is the length of the promoter region. Because the anti-genome has a stronger promoter than the genome [19,31], which is quantified by *S_prom_* in our model, *S_prom_*-fold, more polymerases bind to the promoter of the anti-genome than to that of the genome. Under the limitation of free polymerase complex, the concentration of the polymerases newly binding to the promoters of the genomes or the anti-genomes (*pol_term_^new^*) could be described as follows:

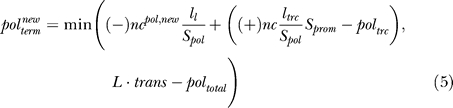
where *S_prom_* is the strength of the anti-genomic promoter relative to that of the genomic promoter, and min(A,B) equals the smaller of A and B. The distribution of newly bound polymerases between genomic and anti-genomic templates is determined by the concentrations of the available free templates of each type and the relative strengths of their promoters:

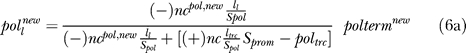


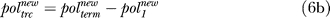
where *pol_trc_^new^* is the concentration of the polymerases newly binding to the complementary trailer region (promoter) of the anti-genome. The polymerases newly binding or already bound to the promoters of the genomes and anti-genomes start viral RNA synthesis as transcription or replication process.

#### Transcription.

The viral polymerase on the leader region of the genome starts either transcription or replication. If there are sufficient N proteins, transcription is inhibited by the encapsidation of nascent positive-sense RNAs by N proteins; then replication dominates transcription [41,52,53]. In contrast, if there are insufficient free N proteins, then transcription dominates replication. In the model we correlate the extent of transcription dominance with the availability of N proteins by introducing a factor, *encap*. This factor is defined as the ratio of the number of free N proteins to the number required to encapsidate all available nascent genome-sized viral RNAs. Only *nocap* (= 1 − *encap*) of the polymerases bound to the genomic promoters can start the transcription:

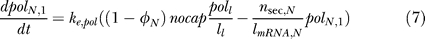
where *pol_N,_*_1_ is the concentration of the polymerases located at the first segment of the N gene (Figure 9C), *k_e,pol_* is the elongation rate of polymerase, *φ_N_* is the attenuation factor for N gene, *n_sec,N_* is the total number of the segments of N gene, and *l_mRNA,N_* is the length of N mRNA (Table 2, [28,29]). The genome segments are continuously charged with incoming polymerases and discharged with outgoing polymerases with a rate of *k_e,pol_* (Equations 7–9). If the polymerase input to the leader region of the genome is decreased owing to a lack of free polymerases, then the polymerase concentrations downstream of the leader region will be subsequently reduced (Figure 9C). There are conserved intergenic sequences involved in letting a fraction of viral polymerases release from the genome template at intergenic sections during transcription, which is so-called partial transcription termination or attenuation (Figure 9C) [18,19]. Because the transcription is initiated from the 3′ end promoter, the attenuation mechanism causes genes more proximal to the 3′ end to be more highly expressed, which ultimately leads to an unequal concentration distribution of viral mRNAs. The extents of partial transcription termination are quantified by the attenuation factors, *φ_i_,* in our model (Figure 9C). These are 0/0.25/0.25/0.25/0.05 for leader–N/N–P/P–M/M–G/G–L intergenic regions, respectively [18,19,26,27]. *φ_i_* fraction of polymerases are released at intergenic region *i*. With Equations 7–9, we simulate the polymerase flux into each gene segment, which is proportional to the elongation rate of polymerase, but inversely proportional to the extent of attenuation:

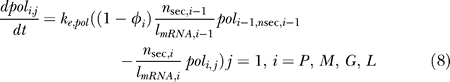


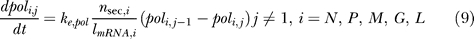
where *pol_i,j_* is the concentration of the polymerases located at the *jth* segment of gene *i,* and *i*−1 indicates the prior gene of gene *i*. The amount of newly synthesized mRNAs for each gene is determined by the concentration of polymerases occupying each gene section on the genome template and the decay rates of the mRNAs:

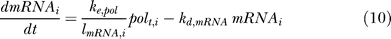
where *mRNA_i_* is concentration of mRNAs for gene *i, k_d,mRNA_* is the decay rate constant of mRNA that is the same for all five viral mRNAs [54], and *pol_t,i_* is the total concentration of the polymerases occupying on the *ith* gene.

Our formulation for transcription assumes that the synthesis of viral mRNAs is rate-controlled by the transcription initiation as well as the elongation of polymerase. Transcription initiation rate is parameterized by the spacing between neighboring polymerases in our model. At a given polymerase elongation rate, the larger polymerase spacing indicates the lower rate of transcription initiation. Transcription initiation modulates the input of polymerases to the leader region of the genome.

#### Translation.

We consider that both translation initiation and polypeptide chain elongation contribute to the rate of viral protein synthesis. The translation initiation rate is parameterized by the ribosomal spacing. In our model we first calculated the number of ribosomes involved in viral translation by considering the maximum concentration of the ribosomes bound to viral mRNAs at a fixed ribosomal spacing:

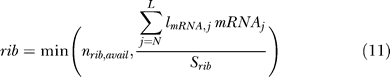
where *rib* and *n_rib,avail_* are the concentrations of the ribosomes actually involved in viral translation and the ribosomes available for viral translation, respectively, and *S_rib_* is the spacing between neighboring ribosomes.

The ribosomes involved in viral translation (*rib*) are allocated to the five types of viral mRNAs according to their length and abundance, assuming that each viral mRNA has the same efficiency of translation initiation [55]:

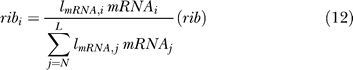
where *rib_i_* is the concentration of the ribosomes assigned to mRNA *i*.

The synthesis rate of each viral protein depends on the elongation rate of the ribosome, linear density of ribosomes on its corresponding mRNA, and its first-order decay rate:

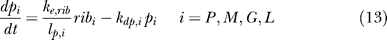
where *p_i_* is the concentration of protein *i, k_e,rib_* is the elongation rate of ribosome, *l_p,i_* is the length of protein *i,* and *k_dp,i_* is the decay rate constant of protein *i*.

We also accounted for the consumption of free N proteins during the encapsidation of genome-length nascent RNAs and assumed that the degradation of nucleocapsids yielded intact N proteins:

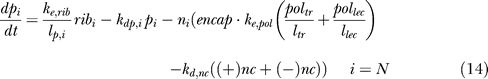
where *n_N_* is the stoichiometry of N protein in a single nucleocapsid or virion progeny (Table 2, [28,29]), *pol_tr_* and *pol_lec_* are the concentrations of the polymerases located on the trailer and complementary leader regions of the genomes and the anti-genomes, respectively, *l_lec_* (= *l_l_*) is the length of the complementary leader region, and *k_d,nc_* is the decay rate constant of nucleocapsid. As progeny virions are assembled, the concentration of each protein is reduced by the amount corresponding to its stoichiometry in a single virion particle.

#### Replication.

We assumed that N protein regulates the switch of the role of polymerase between transcription and replication by encapsidating the newly synthesized RNAs [41,52]**.** The polymerase that starts the replication at the leader region of the genome requires further supply of N proteins to skip the attenuation signals at each gene junction and thereby to complete each round of replication. Depending on the availability of N proteins, *nocap*(*=1-encap*) fraction of polymerases terminate the replication at each gene junction in our model:

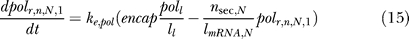
where *pol_r,n,N,_*_1_ is the concentration of the replicating polymerases on the first segment of the N gene section in the negative-sense genomic nucleocapsid.

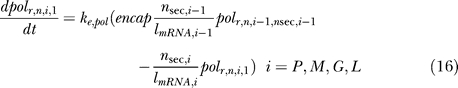
where *pol_r,n,i,_*_1_ and *pol_r,n,i-_*_1*,nsec,i-*1_ are the concentrations of the replicating polymerases on the first segment of gene *i,* and on the last segment of gene *i*−1, respectively.

The level of polymerases that scan through the whole genome (*pol_tr_*) determines the amount of newly synthesized anti-genomic nucleocapsids, (+)*nc*:

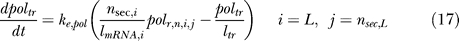


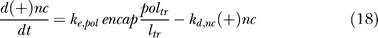
where *l_tr_* (= *l_trc_*) is the length of the trailer region of the genome. We also considered the first-order kinetics for the decay of anti-genomic nucleocapsid.

The synthesis and decay of genomic nucleocapsids are described in the same way as for those of the anti-genomic nucleocapsids except that the polymerases on the anti-genomic templates are not released at intergenic regions:

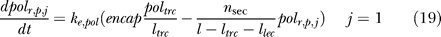
where *pol_r,p,j_* is the concentration of the replicating polymerases on the *jth* segment of the positive-sense anti-genomic nucleocapsids, *l* is the total length of the genome, and *n_sec_* is the total number of segments of the genome (Figure 9B).

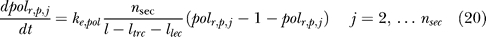


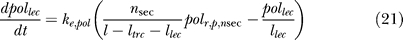


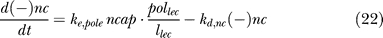



In our model, non-encapsidated nascent genome and anti-genome fragments are released from polymerases and immediately degraded.

As polymerases leave the promoter regions by moving toward the downstream sequences, the concentration of polymerases on the promoters will decrease. The dynamic changes of the polymerase concentrations on the promoters of the genomic and the anti-genomic templates are finally described, respectively:

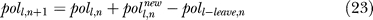
where *pol_l-leave_* is the concentration of the polymerases leaving the genomic promoters, *Ο_,n_* and *Ο_,n+1_* are the concentrations of a component (*Ο*) at time *n* and time *n+*1 (in our numerical integration, time *n+*1 − time *n* = *Δt*), respectively.

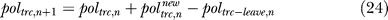
where *pol_trc-leave_* is the concentration of the polymerases leaving the anti-genomic promoters.

#### Assembly and budding.

We assume that the condensation of negative-sense nucleocapsid by M protein initiates the virion assembly and the condensed nucleocapsids are not degraded in the same manner as virion progeny. Whenever the requirement for the stoichiometric amounts of proteins is satisfied, progeny virions are instantaneously assembled and released to the extracellular space. The time required for the condensation of the negative-sense nucleocapsid, the assembly, and the budding of progeny virion was assumed to be negligible relative to the preceding steps.

#### Host cell.

In our model, the host cell provides unlimited building blocks such as nucleoside triphosphates and amino acids for the growth of virus. However, as viral components accumulate during the course of infection, some key host components for translation such as initiation and elongation factors may be depleted [28,63,56]. Two main viral products, leader-mRNA and M protein, contribute to the deficiency by inhibiting the synthesis of host macromolecules at the transcription level [19,28,35,36]. Because leader-mRNA starts to accumulate soon after the initiation of infection, and a small amount of the component is enough to trigger the inhibition [28,35], the pool of host factors is continuously reduced from the onset of infection. We quantify this reduction with a single decay rate constant specific to the type of host cell:

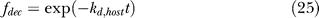
where *f_dec_* is the level of host translation factors at time *t*, relative to that of the initial state of the cell before infection (at *t* = 0), and *k_d,host_* is the decay rate constant.

The inhibition by the leader mRNA causes a first-order decay of the host factors, resulting in a shortage of the ribosomes equipped with the accessory factors for viral translation in the late infection stage in our model. Unlike viral transcription and replication, viral translation is directly affected by the decay of host factors since it depends entirely on host machinery. In the early infection, host mRNAs outnumber viral mRNAs and thereby successfully compete for the host translation machinery. However, the newly synthesized M proteins inhibit the host transcription initiation and the export of host mRNAs from the nucleus to the cytoplasm [57], thereby causing a gradual shift in translation from host mRNAs to viral mRNAs. For our model we assumed that the potency of the inhibition by the M protein was independent of the type of cell and its differentiation state [36], and we developed an empirical formula using available experimental data from the literature [36] to account for the competition between host and viral mRNAs for ribosomes. Lyles at al. cotransfected the host cells with VSV M mRNA and chloramphenicol acetyl transferase (CAT) plasmid DNA, and then they quantified the expression of CAT based on its activity, as a function of the expression of VSV M protein [36]. In their experiment the gene expression of CAT was more reduced at higher M protein expression levels. We assume that the decrease of the expression of CAT (or its activity decrease) is proportional to the decrease of the occupancy of host mRNAs by the translation machinery. Using their experimental data, the occupancy of host mRNAs by the translation machinery is correlated with the number of newly synthesized M proteins in the cytoplasm:

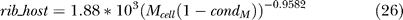
where *rib_host* is the fraction of the translation machinery associated with host mRNAs, and *M_cell_* is the total number of newly synthesized M proteins per cell.

Considering the decay of host factors and the competition between host and viral mRNAs, we could derive a formula to quantify the number of the fully functional ribosomes that are available for the viral protein synthesis over time post-infection (*nrib_avail_*):

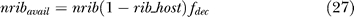
where *nrib* denotes the total concentration of ribosomes whether or not they incorporate all the required accessory factors for their translation function.

Although the ribosomes distribute into membrane-bound and cytoplasmic forms, each class supporting the syntheses of the viral G protein and the other four viral proteins (N, P, M, and L), respectively, we treated the ribosomes in our model as one population.

#### Initial condition for simulation.

The initial condition for our simulation is set by a fixed number of infectious extracellular virus particles per cell (*V_ex_*(0)). At time zero (*t* = 0), the number of bound virus particles and the level of all viral components within cells are zero. In our model, binding of extracellular virus particles to cells reduces their level (Equation 1), and an encapsidated genome and stoichiometric amounts of viral proteins (Table 2, [28,29]) are then immediately released from each bound virus particle to the cytoplasm. Specifically, we assume that all N proteins from a bound virus particle are released as a form of encapsidated genome complex. Downstream processes, beginning with transcription, are then initiated. In our simulation, viral infection starts with *rib_host =* 0.99 (with *rib_host* = 0.9, ~ 0.9995 simulations showed the same results). Other key model parameters for simulation are summarized in Table 1. In addition, a nomenclature list is shown in Table 4.

**Table 4:**
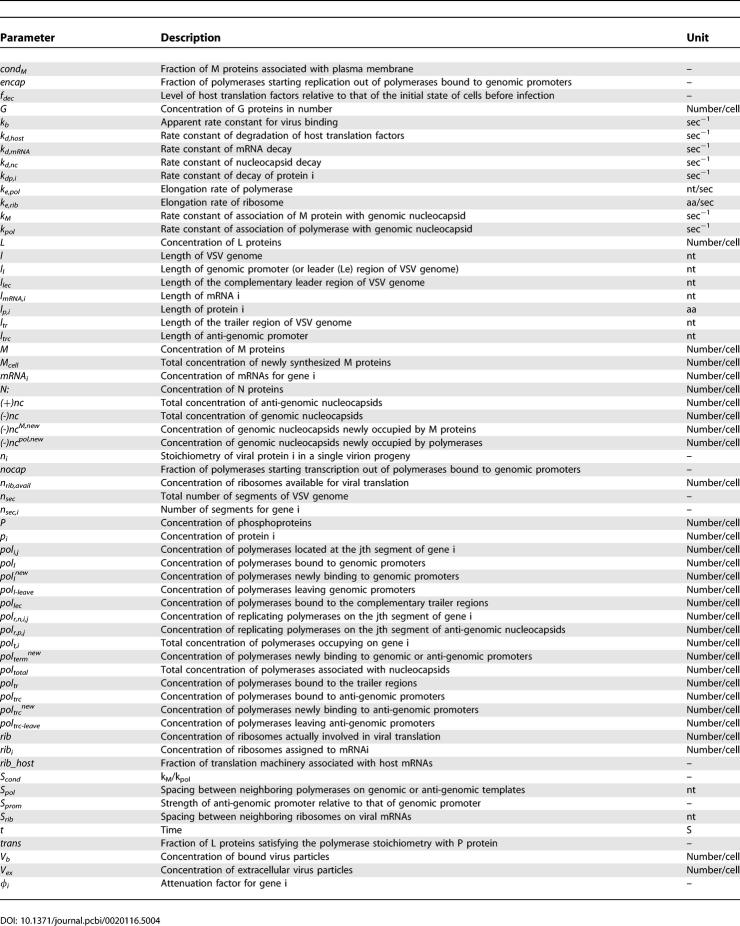
Nomenclature

## Supporting Information

Figure S1 Ratio of Genome to Anti-Genome During Simulated VSV Infection Depends on Relative Promoter Strengths The relative strength of the anti-genomic promoter relative to the genomic promoter is given by *S_prom_*. PI stands for time post-infection. The initial number of infectious extracellular virus particles per cell was three.
(286 KB TIF)

Figure S2 Model Fitting with Experimental Data Two host parameters (*S_rib_* and *k_d,host_*) were obtained by fitting our model with the one-step growth of wild-type virus in BHK or DBT cells (symbols). The simulation results for viral one-step growth are denoted by lines. Initial number of infectious extracellular virus particles per cell was three.
(635 KB TIF)

Figure S3 Flowchart of Iterative Parameter Estimation Process (714 KB TIF)

Figure S4 Productivity Ranking of Six Gene-Shuffled Viruses Provides Parameter Constraint For each value of *S*_*cond*_ and its corresponding host parameter values (*k_d,host_* and *S_rib_*), the virus productivity of each gene-shuffled virus in BHK cells was determined and normalized by the highest productivity. Simulated rankings of six strains matched experimentally observed rankings [9] over a narrow range of *S*_*cond*_ *,* indicated by the bar. Initial number of infectious extracellular virus particles per cell was three.
(872 KB TIF)

Protocol S1 Parameter Estimation

## Abbreviations

aa: amino acids
BHK: baby hamster kidney
CAT: chloramphenicol acetyl transferase
DBT: delayed brain tumor
G: envelope
L: polymerase
M: matrix
N: nucleocapsid
nt: nucleotides
P: phospho
VSV: vesicular stomatitis virus
w/v: weight/volume
