# Supplementary material for: Model-Based Design of Growth-Attenuated Viruses
Source: PLoS Comput Biol. 2006 Sep 1;2(9):e116. doi: 10.1371/journal.pcbi.0020116 (PMC1557587; doi:10.1371/journal.pcbi.0020116)
Supplement: Supplementary file 1 [file pcbi.0020116.sd001.doc]

Protocol S1: Parameter Estimation

Five adjustable parameters in our model were obtained by fitting simulation results to experimental data. Two parameters were specific to the virus: the ratio of rate constants for the association of M protein and polymerase with the genomic nucleocapsid (*S_cond_*) and the strength of the anti-genomic promoter relative to that of the genomic promoter (*S_prom_*) (Eq. (3), (4), (5), (6) and (11) in main text). The remaining three parameters were specific to virus-host interactions or to the host translation machinery: the apparent rate constant of virus binding (*k_b_*) [1], the ribosomal spacing (*S_rib_*), and the decay rate constant of host factors (*k_d,host_*) (Eq. (1), (25) and (27) in main text). Four independent sets of data from the literature and our own experiments were used for the fitting. The parameter estimation was performed by non-linear regression analysis based on the Marquardt and Gauss–Newton methods.

**A.1 Strength of the Anti-genomic Promoter Relative to That of the Genomic Promoter**

In our model the relative abundance of synthesized genome and anti-genome is mainly determined by the strength of the anti-genomic promoter relative to that of the genomic promoter (*S_prom_*) [2-4]. Genomes are produced in excess over anti-genomes [3,5,6]. The degree of excess has been estimated at 20- to 50-fold [7] and 5- to 10-fold [8]. We assumed that the production ratio of genome to anti-genome could vary maximally up to 30. When we assigned 5.4 for the parameter (*S_prom_*), our simulation could satisfy this ratio criterion (Figure S1). Because the production ratio is insensitive to other fitting parameters, compared to the relative strength of the two promoters, we fixed *S_prom_* equal to 5.4 during the subsequent model fittings for the remaining parameters.

**A.2 Association of M Protein and Polymerase with the Genomic Nucleocapsid and Host Parameters**

The parameter that describes the competition between M protein and polymerase for the association with the genomic nucleocapsids (*S_cond_*) is neither easily measured from experiments nor available from the literature. We estimated it indirectly by fitting our model with the growth ranking of gene-shuffled strains that was experimentally observed by others. Here we assume the biophysical parameters that describe interactions among different gene products are independent of their timing or level of expression. Ball et al. constructed six gene-shuffled VSV strains from cDNA of VSV genome, and the strains were ranked in growth, based on the maximum virion production of host cells infected by individual virus strain, from the highest to the lowest as follows: 3’-N-M-P-G-L-5’ (MPG), 3’-N-M-G-P-L-5’ (MGP), 3’-N-P-M-G-L-5’ (PMG, wild type), 3’-N-G-M-P-L-5’ (GMP), 3’-N-P-G-M-L-5’ (PGM), 3’-N-G-P-M-L-5’ (GPM) [9]. The growth experiments were performed using monkey kidney cells (BSC-1) as hosts. In our simulations the relative growth of the six VSV strains was mainly determined by the rate constant ratio of the associations of M protein and polymerase with the genomic nucleocapsids (*S_cond_*). We assumed that the same growth ranking is maintained in our kidney cell line BHK, where we have experimental data for the one-step growth of wild type VSV (Figure S2). Given various *S_cond_* values, we obtained the corresponding values for the host parameters such as decay rate constant of host factors (*k_d,host_*) and ribosomal spacing (*S_rib_*) (Eq. (11), Eq. (25) and Eq. (27) in main text) by fitting our model to the one-step growth data of wild type (wt) virus (PMG) (Figure S3). Here, the objective function used for the data fitting was minimization of normalized deviation between simulated data points and experimental data points (Figure S2):

where *t* is a time point post infection when the number of produced viruses was experimentally measured or computationally predicted. Components of vectors, *y_observed, t_* and *y_predicted, t_* are triplicate data from the measurement and the prediction for time *t*, respectively. is the averaged value of components of *y_observed, t_*.

With each set of parameter values we further simulated the growth dynamics of the other five strains, and then we ranked the six strains including wild type based on their infected cell yields of virus progeny (Figure S4). Only within a narrow range of the parameter (*S_cond_*), could the simulation satisfy the experimentally observed ranking (Figure S4). Choosing the arithmetic average of the lowest and the highest values of the range, we then refitted our model to the one-step growth data of wild type virus and re-estimated the host parameters (Figure S2). The ultimately estimated ribosomal spacing (=238.5 nt) is comparable to that of Chinese Hamster Ovary (CHO) cell (= 300 nt), which we calculated from the experimentally estimated number of ribosomes on the VSV mRNAs [10].

Depending on the host type, there can be variations in the availability of host translation factors and their decay rates. Assuming that *S_cond_* is an intrinsic property of VSV self-regulation, independent of virus-host interactions, we estimated host parameters for DBT cells by fitting our model with one-step growth of wt VSV in DBT cells (Figure S2 and Table 1). Due to the slower decay rate of translation factors in DBT cells, the virion production continued for longer time period than in BHK cells. All the parameters obtained from the fitting process and the literature are summarized in Table 1.

**REFERENCES**

1. Lim KI (2005) Dynamic mapping from virus genotype to growth phenotype. Ph.D. thesis: University of Wisconsin-Madison.

2. Finke S, Conzelmann KK (1997) Ambisense gene expression from recombinant rabies virus: random packaging of positive- and negative-strand ribonucleoprotein complexes into rabies virions. J Virol 71: 7281-7288.

3. Le Mercier P, Garcin D, Hausmann S, Kolakofsky D (2002) Ambisense sendai viruses are inherently unstable but are useful to study viral RNA synthesis. J Virol 76: 5492-5502.

4. Le Mercier P, Garcin D, Garcia E, Kolakofsky D (2003) Competition between the Sendai virus N mRNA start site and the genome 3'-end promoter for viral RNA polymerase. J Virol 77: 9147-9155.

5. Plumet S, Duprex WP, Gerlier D (2005) Dynamics of viral RNA synthesis during measles virus infection. J Virol 79: 6900-6908.

6. Simonsen CC, Batt-Humphries S, Summers DF (1979) RNA synthesis of vesicular stomatitis virus-infected cells: in vivo regulation of replication. J Virol 31: 124-132.

7. Rose JK, Whitt MA (2001) Rhabdoviridae: The viruses and their replication. Philadelphia: Lippincott Williams & Wilkins.

8. Wagner RR, Rose JK (1996) Rhabdoviridae: The viruses and their replication. Philadelphia: Lippincott Williams & Wilkins.

9. Ball LA, Pringle CR, Flanagan B, Perepelitsa VP, Wertz GW (1999) Phenotypic consequences of rearranging the P, M, and G genes of vesicular stomatitis virus. J Virol 73: 4705-4712.

10. Lodish HF, Froshauer S (1977) Rates of initiation of protein synthesis by two purified species of vesicular stomatitis virus messenger RNA. J Biol Chem 252: 8804-8811.

**FIGURE LEGENDS**

Figure S1. The production ratio of genome and anti-genome during VSV infection as function of *Sprom*. PI stands for post infection. Initial number of infectious extracellular virus particles per cell was three.

Figure S2. Model fitting with the experimental data. Two host parameters (*S_rib_* and *k_d,host_*) were obtained by fitting our model with the growth of wild type virus in BHK or DBT cells (symbols). The simulation results for viral growth are denoted by lines. Initial number of infectious extracellular virus particles per cell was three.

Figure S3. Flowchart of iterative parameter estimation process

Figure S4. The relative virion production of the six gene-shuffled virus strains as function of *Scond*. At each value of *Scond* and its corresponding host parameter values (*k_d,host_* and *S_rib_*) the virion production of each gene-shuffled virus in BHK cells is normalized by that of the fittest one. Therefore, the fittest one has the relative virion production of 1 over the range of the parameter. Only within a narrow range of the parameter covered by the bar, simulations could match with the experimentally observed growth ranking of the six strains [9]. Initial number of infectious extracellular virus particles per cell was three.
